# Assessing knowledge of herbal medicine course for dental students

**DOI:** 10.1186/s12906-022-03801-z

**Published:** 2022-12-03

**Authors:** Zuhair S. Natto

**Affiliations:** 1 Department of Dental Public Health, Faculty of Dentistry, King Abdulaziz University, Jeddah, Saudi Arabia; 2 Department of Periodontology, Tufts University School of Dental Medicine, MA Boston, USA; 3 Department of Oral Health Policy and Epidemiology, Harvard University School of Dental medicine, MA Boston, USA

**Keywords:** Herbal medicine, Dentistry, Course, Knowledge, Education

## Abstract

**Background:**

The aims of this article are to assess dental students’ knowledge about herbal medicine usage and the potential benefits and side effects, and to conduct a short course about herbal medicine.

**Methods:**

All fourth-year pre-doctoral students were invited to participate in a herbal medicine course as a test while the sixth-year students were the control group. A survey was tested for validity and reliability. It comprised of 16 multiple choice questions was given before the course and one month after the course. The sum score of knowledge for each participant was calcuated based on the ability to identify the use of herbs in dentistry with high-quality evidence (correct answer) or total answer for periodontal disease and caries.

**Results:**

The response rate for completing the study was 112 fourth-year students (73.7%) and 64 sixth-year students (39.0%). More than half of the participants (52.5%) were unsure about the importance of herbal medicine in dentistry. However, the majority also stated that the most common herbs used in dentistry were clove (62.9%), followed by curcuma turmeric (54.7%) and meswak (43.0%). The fourth-year students displayed evidence of a higher overall knowledge score after the course in herbal medicine related to periodontal disease in total and correct answers (mean 4.48 ± 4.13, 3.73 ± 3.31, respectively) compared to before the course (mean 0.84 ± 1.23, 0.74 ± 1.16, respectively) (p-value < 0.001). The post-course periodontal disease total and correct answers were statistically significant between fourth- and sixth-year students.

**Conclusion:**

Herbal medicine has a potential positive impact on dentistry. However, these effects are not fully investigated and received full attention in academic institute. This short educational program related to medicinal herbs can improve the knowledge of dental students. This will help increase the awareness about the use and potential side effects of herbal medicine in the dental field.

## Background

The use of herbal medicine/supplements is popular throughout the world, including developed countries [1–4]. In 2007, 17.7% of the United States’ adult population had used some form of herbal supplement [5], spending about $15 billion on them [5]. These supplements are also known as “non-vitamin, non-mineral, natural products” [5]. Many individuals take them for disease treatment and pain related to the back (17.1%), neck (5.9%), joints (5.2%), and arthritis (3.5%)[6]. Most users were females and above 40 years old [6].

Herbal medicine is part of the complementary and alternative medicine (CAM) field [1–4, 7, 8], which focuses on the treatments and procedures administered alongside or instead of conventional therapy [1–4, 7, 8]. CAM is divided into three groups: (a) natural products; (b) mind and body practices; and (c) other complementary health approaches [1, 2, 7, 8].

Thus far, most research on the knowledge and perception has focused on adults or students [9–12]. There have also been several studies conducted on medical students [9–12]. However, very limited research has been done among the dental students. Moreover, their usage, potential benefits, and side effects have not been confirmed. Hence, it was suggested to conduct a short course about herbal medicine based on the optimal available evidence on this topic.So, the aim of this study to present another effort to improve dentists’ knowledge through the introduction of specialized course in dental herbs medicine at faculty of dentistry at King Abdulaziz University (KAUFD). The course’s goal is to increase the dental students knowledge, resulting in better dental care, and to produce dentists equipped with the necessary awareness about dental herbal medicine.

## Methods

### Hypothesis:

After the course, the dental students will have expanded their knowledge of the uses of herbal medicine in dentistry compared to before the course.

### Intervention:

King Abdulaziz University Faculty of Dentistry (KAUFD) developed a herbal medicine course to improve students’ knowledge about herbal medicine uses, impacts, and side effects. The course, which lasts a total of four hours, is held over two days in the Fall semester. It is optional and has a didactic component only in terms of lectures and discussion. The predoctoral program lasts six years in total; the herbal medicine course was introduced for fourth-year students, which is their first year of clinical practice.

### Study Design:

**Fig. 1:**
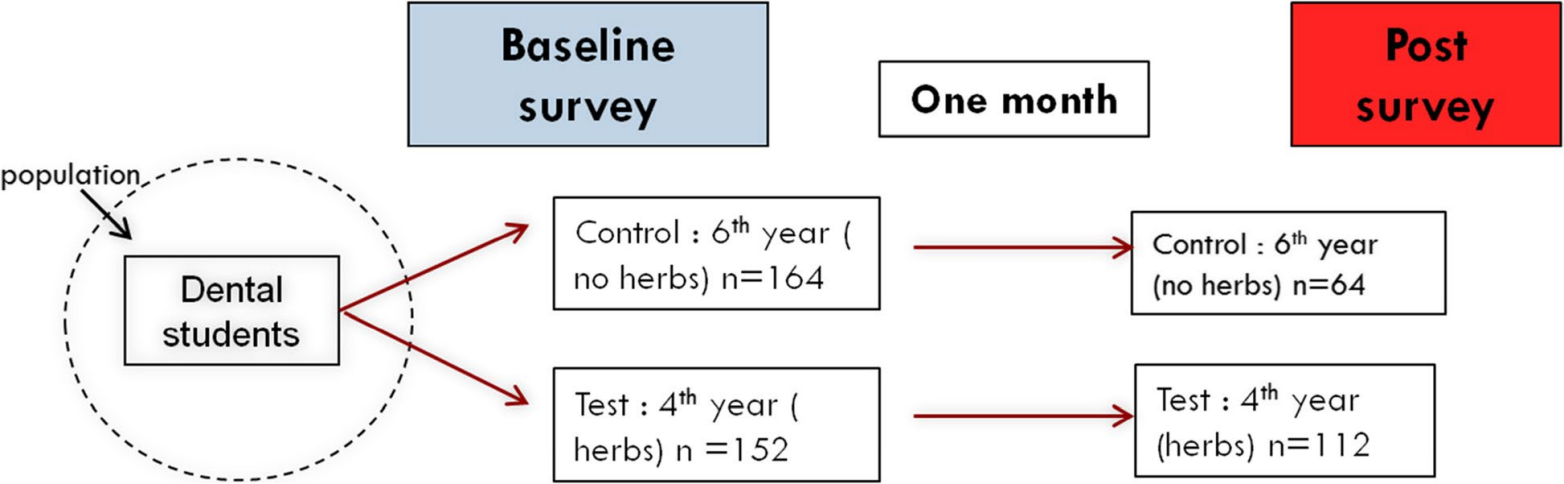
Flowchart of the study design

The study consisted of two groups: fourth-year students, who were invited to take the course as a test group, and the sixth-year students, who did not take the course as a control (Fig. 1). Neither group were informed at the beginning whether they were in the test or control group. The study was approved by the ethical committee at KAUFD (# 295-10-21).

The lecture was created according to the evidence-based dentistry concept of what has been published in herbal medicine [13–17]. In another way, more credit was given to high-quality meta-analysis, systematic review, randomized clinical trials, and cohort, while less credit was given to low-quality cross-sectional studies and reviews.

### Survey validation and relibilaity

A survey containing 16 multiple-choice questions, and which included an “I don’t know” option for several questions, was distributed before and one month after the course. A survey questions was then established based on the materials that will be covered in the lecture. The survey was tested for content validity and distributed to 8 individuals with expertise in some aspect of the subject matter. These individuals included herbal medicine experts and dental consultants in oral medicine, restorative dentistry, periodontics, and maxillofacial surgery. They were asked to check and rate the importance of each question using a five-point Likert scale (from 1 = very important to 5 = not important). They determined if an item should be included in the questionnaire. For face validity, the same survey was reviewed by four different fifth-year students to confirm the clarity of the questions before distribution. They repeated the survey a second time after a one-week period. The results were compared for consistency by calculating kappa statistics which was ranged between 0.78 and 0.86. The internal consistency (Cronbach’s alpha coefficient ) was 0.738 (95% confidence interval [CI]: 0.671–0.795) for periodontal disease and 0.806 (95% CI: 0.757–0.848) for caries. We modified the web survey based on the results of face and content testing, as well as the results of the reliability testing.

Questions were related to: (1) demographic such as gender, level, and marital status (4 questions); (2) personal opinion and use of herbal medicine (7 questions ); (3) herbal medicine and dentistry (5 questions). Participants understood that the course was voluntary and would not be graded. The participating students also completed an electronic consent form. Student ID number was used as the link between the two surveys.

### Sample size and data analysis :

This is a pilot study as we did not find a similar study design in the dental field. It was a convenience sampling of fourth and sixth -year students who voluntarily participated in this study. All data were entered and analyzed using SPSS version 21. Responses of each question were summarized to create an overall frequency and percentage. The sum score of knowledge for each participant was calcuated based on the ability to identify the potential use of herbs in dentistry with high-quality evidence (correct answer) or total answer for periodontal disease and caries. To do this step, first we identified all the systematic review, clinical trial and observational study (cohort and case control studies only ) articles which linked between herbs and dentistry. Then, identified if a herb could be potentially benefical in periodontal disease disease or caries or not at all. These answers were considered the correct answers. We analyzed all the students answers (total) including incorrect answers. This was analyzed using the Wilcoxon signed-rank test. A significant difference is described as a *p* value less than 0.05.

## Results

### Description of the sample:

The pre-course survey was sent to 316 students (152 fourth-year and 164 sixth-year students) and the response rate was 100%. However, the students who agreed to take the course comprised 114 students among the fourth-year respondents (74.0%). Out of the total fourth- and sixth-year students, those who answered the post-course survey comprised 112 fourth-year students (73.7%) and 64 sixth-year students (39.0%) ( Fig. 1). There were 37 females and 27 males among the sixth-year students and 61 females and 53 males among the fourth-year students. Only two students were married; both were female, and each was from each year group .

### Overall personal opinion and use of herbal medicine:

The fourth-year students ( test group ) displayed evidence of a higher overall knowledge score after the course in herbal medicine related to periodontal disease in total and correct answers (mean 4.48 ± 4.13, 3.73 ± 3.31, respectively) compared to before the course (mean 0.84 ± 1.23, 0.74 ± 1.16, respectively) (*p*-value < 0.001). The post-course periodontal disease total (4.48 ± 4.13 vs. 0.59 ± 0.87 ) and correct answers (3.73 ± 3.31 vs. 0.52 ± 0.79 ) were statistically significant between fourth (test)- and sixth (control)-year students (*P*-value < 0.001).

### Individual personal opinion and use of herbal medicine:

The majority of the participants believed that it is beneficial to use herbal medicine (85.4%) and had not noticed any side effects during personal use or other use (93.7%) (Table 1). However, only 69.6% of them had previously used herbal medicine. Among those who had not yet used it, the main reasons for not using it were “do not know much about it” (77.1%) followed by “no reason” (55.2%). The main sources of the information regarding herbal medicine were elderly relatives (77.8%), followed by Internet (51.6%) and friends (44.3%). Most agreed that their patients should tell the physician if they used it (90.5%). However, some barriers were identified, such as insufficient scientific evidence (62.7%) and lack of trained professionals (37.0%) (Table 2). Among those who had used herbal medicine before, ginger was the most commonly used remedy (63.7%) followed by green tea (54.0%), cinnamon (42.7%), and black seed (43.3%) (Table 2).

**Table 1:** Personal opinion and use of herbal medicine of all study sample

Variable	N (%) 316
Benefit to use	
Beneficial	270(85.4)
Placebo	46(14.6)
Did you use	
Yes	220(69.6)
No	96(30.4)
Any side effect	
No	296(93.7)
Yes	20(6.3)
The reason of not use :	
Do not know much about it	74(77.1)
No reason	53(55.2)
Do not need it	40(41.7)
Others	26(27.1)
It is not safe to use	18(18.8)
Not effective	17(17.7)
Source of herbs information	
Elderly	246(77.8)
Internet	163(51.6)
Friend	140(44.3)
Lecture class	29(9.2)
Textbook	28(8.9)
TV	27(8.5)
Labels on product container	13(4.1)
Inform the doctor about herbal use	
Yes	286(90.5)
No	30(9.5)
Barrier to use herbal medicine	
Insufficient scientific evidence	198 (62.7)
Lack of trained professional	117(37.0)
No reason	63(19.9)
Insufficient education	61(19.3)
It is not safe to use	50(15.8)

**Table 2:** The most common herbs used among the dental students participants who used before any type of herbs

What did you use	n (%) 220
Ginger	140(63.7)
Green tea	119(54.0)
Cinnamon	94(42.7)
Black seed (Nigella sativa)	93(43.3)
Anise plant	89(40.5)
Peppermint	88(40.0)
Commiphora myrrha	84(38.2)
Chamomile	83(37.7)
Meswak	74(33.6)
Garlic	64(29.1)
Clove	59(26.8)
Curcuma (Turmeric)	43(19.5)
Aloevera	42(19.1)
Castor oil	37(16.8)
Fenugreek	34(15.5)
Fennel flower	19(8.6)
Bran	16(7.3)
Ginseng	6(2.7)

### Herbal medicine and dentistry:

More than half of the participants were unsure about the importance of herbal medicine in dentistry (52.5%). (Table 3). However, they mentioned that the most common herbs used in dentistry were clove (62.9%), followed by curcuma turmeric (54.7%) and meswak (43.0%) (Table 3).

**Table 3:** The most common herbs used in dentistry

Variables	N (%) 316
Herbs important in dentistry?	
Maybe	166(52.5)
Yes	81(25.6)
No	69(21.8)
The most common herbs in the dental field	
Clove	199(62.9)
Curcuma (Turmeric)	173(54.7)
Meswak	136(43.0)
Commiphora myrrha	52(16.5)
Cinnamon	48(15.2)
Green tea	29(9.2)
Peppermint	27(8.5)
Ginger	23(7.3)
Fennel flower	22(6.9)
Chamomile	17(5.4)
Fenugreek	16(5.1)
Anise plant	15(4.7)
Garlic	13(4.1)
Castor oil	4(1.3)
Neem	4(1.3)
Aloevera	3(0.9)
Ginseng	1(0.3)
Bran	1(0.3)

Meswak and commiphora myrrha were the most commonly mentioned remedies for periodontal disease before and after the lecture, while clove and meswak were the most common herbs mentioned for use in caries prevention among the test group (Table 4). The answers were almost identical for the control group; however, the percentages were much lower (Table 5).

**Table 4:** The most common herbs used in caries prevention and periodontal disease among the test group (herbs course)

The most common herbs for periodontal disease before the course n (%) 112	The most common herbs for periodontal disease after the course n (%) 112	The most common herbs for caries before the course n (%) 112	The most common herbs for caries after the course n (%) 112
Meswak 26(23.2)	Meswak 47(41.9)	Clove 50(44.6)	Clove 37(33.0)
Commiphora myrrha 18(16.1)	Commiphora myrrha 51(45.5)	Meswak 35(31.3)	Meswak 18(16.1)
Clove 16(14.3)	Chamomile 38(33.9)	Cinnamon 22(19.6)	Green tea 11(9.8)
Peppermint 6(5.4)	Clove 35(31.3)	Green tea 19(16.9)	Peppermint 10(8.9)
Cinnamon 6(5.4)	Green tea 34(30.4)	Peppermint 19(16.9)	Cinnamon 8(7.1)
Green tea 4(3.6)	Cinnamon 27(24.1)	Ginger 15(13.4)	Chamomile 7(6.3)
Chamomile 3(2.7)	Aloevera 27(24.1)	Chamomile 11(9.8)	Ginger 7(6.3)
Garlic 2(1.8)	Ginger 25(22.3)	Garlic 10(8.9)	Commiphora myrrha 6(5.4)
Curcuma (Turmeric) 4(3.6)	Garlic 23(20.5)	Fenugreek9(8.0)	Anise plant 6(5.4)
Ginger 3(2.7)	Curcuma (Turmeric) 22(19.6)	Curcuma (Turmeric) 8 (7.1)	Garlic 5(4.5)
	Anise plant 20(17.9)	Anise plant 5(4.5)	Curcuma (Turmeric) 3(2.7)
Fenugreek1 (0.9)	Peppermint 18(16.1)	Commiphora myrrha 3(2.7)	Castor oil 2(1.8)
Anise plant 1(0.9)	Fenugreek17(15.2)	Aloevera 1(0.9)	Aloevera 1(0.9)
Aloevera 1(0.9)	Castor oil 8(7.1)		Fenugreek1(0.9)
	Ginseng 5(4.5)		Ginseng 1(0.9)
I do not know 61(54.5)	I do not know 47 (41.9)	I do not know 12(10.7)	I do not know 48 (42.9)

**Table 5:** The most common herbs used in caries prevention and periodontal disease among the control group ( no herbs course)

The most common herbs for periodontal disease before the course n (%) 64	The most common herbs for periodontal disease after the course n (%) 64	The most common herbs for caries before the course n (%) 64	The most common herbs for caries after the course n (%) 64
Meswak 16(23.6)	Meswak 15(34.8)	Clove 9(33.1)	Clove 19(31.5)
Commiphora myrrha 17(19.7)	Clove 20(30.9)	Meswak 6(23.0)	Meswak 10(15.7)
Clove 15(17.4)	Commiphora myrrha 2(29.8)	Commiphora myrrha 9(6.7)	Peppermint 6(8.9)
Peppermint 5(6.2)	Green tea 5(21.9)	Fenugreek2(6.2)	Cinnamon 6(7.9)
Cinnamon 4(5.6)	Chamomile 1(21.9)	Chamomile 1(6.7)	Commiphora myrrha 5(6.2)
Green tea 4(4.5)	Cinnamon 9(20.2)	Cinnamon 1(12.9)	Curcuma (Turmeric) 3(5.6)
Chamomile 4(3.9)	Ginger 3(15.7)	Anise plant 1(3.8)	Chamomile 3(5.6)
Garlic 3(2.8)	Garlic 3(14.6)	Ginseng 2(1.1)	Ginger 2(5.1)
Castor oil 3(1.9)	Curcuma (Turmeric) (12.9)	Aloevera 1(1.1)	Garlic 2(3.9)
Fenugreek2 (1.9)	Peppermint 5(12.9)	Castor oil 2(1.1)	Green tea 2(7.3)
Curcuma (Turmeric) 1(2.8	Fenugreek3(11.2)	Bran1(0.6)	Aloevera 1(1.1)
Ginger 1(2.2)	Castor oil 1(5.1)		Anise plant 1(3.9)
Anise plant 1(1.1)	Bran1(0.6)		
Bran1(0.6)			
I do not know 38 (59.4)	I do not know 29 (45.3)	I do not know 40 (62.5)	I do not know 28 (43.8)

### Sum score of knowledge:

There was a significant improvement was seen in all subjects’ total knowledge regarding periodontal herbs ( mean of answers was 0.83 before the program and 3.09 after one month) ( *p* < 0.001) (Table 6). The mean of the correct answer was increased significantly as well (0.70, and 1.58, respectively, *p* < 0.001 ± 3.09 ). The sum score of knowledge of total and correct answers regarding caries was higher after the program. However, it was not statistical significant (Table 6).

**Table 6:** The sum score of knowledge based in the ability to identify the herbs with high-quality evidence (correct answer) or total answer for periodontal disease and caries

Variables	Total periodontal hebrs	Correct periodontal hebrs	Total caries hebrs	Correct caries hebrs
Herbs course				
Baseline	0.84 ± 1.23	0.74 ± 1.16	1.24 ± 1.81	1.00 ± 1.32
At follow up	4.48 ± 4.13	3.73 ± 3.31	1.68 ± 2.63	1.32 ± 1.87
*P* value	< 0.001*	< 0.001*	0.225	0.210
No herbs course				
Baseline	0.79 ± 1.27	0.64 ± 0.98	1.21 ± 1.63	1.03 ± 1.35
At follow up	0.59 ± 0.87	0.52 ± 0.79	1.03 ± 1.41	0.79 ± 1.04
*P* value	0.304	0.383	0.646	0.275
Total				
Baseline	0.83 ± 1.24	0.70 ± 1.09	1.16 ± 1.67	0.92 ± 1.23
At follow up	3.09 ± 3.08	1.58 ± 3.09	1.51 ± 2.32	1.22 ± 1.70
*P* value	< 0.001*	< 0.001*	0.210	0.095

*P value < 0.001

## Discussion

Several studies have been conducted to assess the perceptions and knowledge of different population groups regarding herbs [18, 19]. However, few articles have been conducted in the dental field [20–22]. In the medical field, Yeo et al. found that 92% of medical students believed that conventional medicine would be more effective than herbs [9]. In our study, 85.4% of participating dental students believed that herbs are beneficial. Their main sources of information are the elderly, the Internet, and friends.

In the dental field, only 25% believed that herbal medicine is beneficial and the remainder were either unsure or did not think. The main reasons were insufficient scientific evidence and lack of trained professionals, which is in agreement with Harris et al., who found that 53.0% of students did not prefer to use herbal therapies if there is no scientific evidence to support them [23].

Most oral health issues were associated with bacterial plaque, the removal of which will improve oral health [24, 25]. Mechanical or chemical removal of plaque is most common. One chemical method is the mouth rinse, which can prevent or facilitate the plaque removal; these treatments are an adjunct method, and herbal medicine was introduced and experimented in different forms to prevent the development of plaque chemically [24, 25].

Several published articles demonstrate the potential benefits of herbal medicine against dental caries and periodontal disease [15]. These treatments/preventions could be safer due to reduced toxicity and cheaper than chemical drugs. For example, neem, eucalyptus, tulsi, and clove have antibacterial properties, which can help to treat forms of gingival inflammation [16]. Herbal medicine is useful in several cases such as caries prevention, toothache, mouth ulcers, gingivitis, mouth ulcers, oral thrush, and hairy tongue [14]. Some of these herbs have been recommended even for titanium implant coating such as Malus Domestica (apple), and for periodontal filler in periodontal regeneration such as Cissusquadrangularis (veldt grape) and Carthamustinctorius (safflower) [17].

Arabic culture overall has a strong belief in herbal medicine, due to its significant historical background in traditional Arab medicine [26]. However, the current research in this field in the Arabic region is small, with limited up-to-date knowledge on the Arabic forms of herbs [27, 28]. According to a survey, out of the 2,600 plant species in the Middle East, more than 700 plant are known to be used as medicinal herbs. Currently, traditional Arab medicine uses less than 200–250 plant species for the treatment of multiple diseases [28–39]. The current status of Arab herbal medicine is concerning, because it is not part of the curriculum nor has any specific academic programs to support. In other countries, India has 57 universities and research institutes that focus on traditional medicine, while South Korea has established 12 universities and institutes focusing on traditional Korean medicine [29].

A possible limitation of this research is that only about 75% of the participants took the course. We are not sure about the grade level of the students who did not participate and we did not evaluate whether the course would cause any burden to their studies. Another problem is that the students knew the course was not graded, and thus may not have taken it seriously. Moreover, we are comparing between fourth-year students (test) and sixth-year students (control), which could create bias because the sixth-year students have been exposed to more dental materials/patients and may be more aware of herbal medicine. However, the baseline analysis did not reveal any significant difference between them. This course did not evaluate the family background of the participants which might affect the results. Another limitation is the drop out in the control group (60%) which might affect the results, and there was a chance for the control group to read more about herbal medicine and educate themselves before the follow up. However, they did not know the actual date of the follow up, and there was no significant results when we compared knowledge changes in the control group. In addition, this is the first course and we are expecting to face these difficulties and we tried to encourage and remind the students to participate in this research.

## Conclusion

Herbal medicine has a potentially positive impact on dentistry. However, these effects have not been fully investigated and have received insufficient attention from academic institutions. A short educational program on medicinal herbs provided to dental students can improve their knowledge of the field. This will help increase awareness about the use and potential side effects of herbal medicine. However, further investigation is necessary to assess the long-term effects of the program.

## Data Availability

The datasets generated and/or analyzed during the current study are not publicly available due to privacy but are available from the corresponding author on reasonable request.

## Abbreviations

CAM: Complementary and alternative medicine
KAUFD: King Abdulaziz university faculty of dentistry
